# How to Vote

**Published:** 1914-05-09

**Authors:** 


### How to Vote.

Voters must record in the sections reserved for the purpose the distinctive letter appearing at the top of each description. For example, if you consider A to be the best ward locker, you will insert this letter in Section 1, and so on, placing all six descriptions in the order which you consider the merits of each warrant. When filled in, signed, and dated the coupon should be cut out, placed in an envelope, and forwarded to The Editor, The Hospital, 28 and 29 Southampton Street, Strand, London, W.C., marked "Ward Locker Vote" in the top left-hand corner. **All voting papers must reach this office not later than Saturday May 16, 1914.**

